# YOLO-MDEW:Improved YOLOv8 for application of wood board edge banding defect detection

**DOI:** 10.1371/journal.pone.0348758

**Published:** 2026-05-08

**Authors:** Enjing Xiao, Rougang Zhou, Zhenchao Ruan, Chou Jay Tsai Chien, Junjie Zhu

**Affiliations:** 1 School of Mechanical Engineering, Hangzhou Dianzi University, Hangzhou, China; 2 Wenzhou Institute of Hangzhou Dianzi University, Wenzhou, China; 3 Mstar Technologies, Inc., Hangzhou, China; 4 Shanghai Starriver Bilingual School, Shanghai, China; 5 Chongqing Railway Transport Technician College, Shapingba, Chongqing, China; University of Sharjah, UNITED ARAB EMIRATES

## Abstract

With the growing demand for wooden furniture, accurate and efficient detection of edge banding defects in wood panels has become increasingly important. To address the limitations of existing methods—such as low accuracy, high missed detection rates, and frequent false positives—this study proposes an improved YOLOv8-based detection algorithm, termed YOLO-MDEW. Built upon the YOLOv8n framework, the model integrates several key enhancements: the C2f-MCFF(Multi-Channel Feature Fusion) module replaces the original C2f structure to improve multi-scale feature extraction; an enhanced SPPF-D(Spatial Pyramid Pooling with Dilation) module is incorporated to strengthen cross-scale information fusion; and an Efficient Local Attention (ELA) mechanism is applied within SPPF-D to better capture fine-grained defect features. Additionally, the original CIoU loss is replaced with Wise-IoU v3 (WIoU) to accelerate convergence and improve localization accuracy. Experimental results on a custom-built wood panel edge defect dataset demonstrate that YOLO-MDEW achieves a mean average precision (mAP) of 74.0%, representing a 1.9% improvement over the baseline YOLOv8n. These results highlight the proposed method’s enhanced robustness and effectiveness in detecting edge banding defects in wooden panels.

## Introduction

With the continuous advancement of the economy and society, the demand for furniture has steadily increased, driving consistent growth in the annual market for wooden furniture. According to recent statistics [1], China’s furniture industry achieved an operating income of 677.15 billion yuan in 2024, marking a year-on-year growth of 0.4%. Additionally, cumulative furniture output rose by 8.4% year-on-year. On the international front, furniture exports reached USD 67.88 billion in 2024, reflecting a 5.8% increase [1]. These figures highlight the vast scale and substantial growth potential of the furniture industry.

Owing to their environmental friendliness, aesthetic value, durability, and cost-effectiveness, wooden furniture products are increasingly favored by consumers [2]. As the core material in furniture manufacturing, wood-based panels play a critical role in determining the structural and visual quality of final products.

Defect detection in wood-based panels typically involves recognition, classification, and localization of defects [3]. Among these, edge banding is a vital process step that directly affects both functionality and appearance. High-quality edge banding not only improves visual appeal but also minimizes the release of volatile organic compounds (e.g., formaldehyde) and enhances environmental compliance [4,5]. However, various factors—such as processing inconsistencies and material heterogeneity—frequently result in edge defects [6].

With rising quality standards, manufacturers face increasingly stringent demands on inspection and quality control. Currently, many production lines still rely on manual inspection, which suffers from inefficiency, high error rates, and subjectivity. Therefore, there is an urgent need to develop automated and intelligent defect detection methods tailored for edge banding inspection. The emergence of deep learning and convolutional neural networks (CNNs) has revolutionized the field of object detection. These algorithms are generally categorized into single-stage and two-stage detectors. Single-stage detectors, such as SSD [7], RetinaNet, and the YOLO (You Only Look Once) family [8–10], generate predictions in a single forward pass and are known for their real-time efficiency. In contrast, two-stage detectors, such as the R-CNN series [11,12], first generate region proposals and then refine predictions, typically achieving higher accuracy at the cost of speed and computational overhead.

Owing to their robustness and adaptability to complex visual scenarios, CNN-based approaches have seen increasing application in wood panel defect detection [13–15]. For example, Cui et al. proposed a cascaded ELAN network (C-ELAN), introducing a fast supervised attention module to improve small defect recognition [16]. Zhou et al. incorporated a hybrid attention module and dilated convolutions to mitigate feature loss [17]. Ghost convolutions were further applied to reduce parameters without sacrificing performance.

Despite these advancements, research on edge defect detection remains relatively scarce.In addition to CNN-based approaches, recent studies have explored advanced computational methods in related domains.For example, graph attention mechanisms combined with reinforcement learning have been applied to model complex interactions in autonomous driving systems, achieving improved decision-making performance [18]. In signal processing, improved mode decomposition techniques have been proposed for vibration signal denoising, effectively preserving useful information while reducing noise [19]. Furthermore, neural network-based proxy models have been utilized in topology optimization tasks, demonstrating strong capability in solving complex engineering problems [20]. These representative studies highlight the effectiveness of advanced learning-based methods in handling complex and multi-scale problems across different domains. Chen et al. [21] addressed blunt defect detection using a hybrid approach combining Gabor filters, structured light, and InceptionResNetV2, but the system only covered limited defect types. Du Xingyu [22] applied SRGAN to enhance training samples and integrated SENet into YOLOv3, yet the dataset scale and defect diversity remained insufficient. Similarly, He Han [23] added an AW-CBAM attention module to YOLOv5 to improve small-object detection, though scalability was not extensively validated.

In practical production environments, wood panels are produced in large volumes and wide variations, with edge defects that differ in location, shape, and scale. The frequent presence of small or overlapping defects presents major challenges for accurate detection. This study aims to address these challenges by proposing an enhanced YOLOv8-based edge defect detection algorithm tailored to handle complex, small-scale, and diverse defect instances more effectively.The key contributions of this paper are summarized as follows:

1. A new MCFF module is designed to improve the C2f structure, enhancing the model’s feature extraction capability through multi-branch convolutional information fusion.
2. Based on the original SPPF module, average pooling is introduced to enhance its structure, thereby improving the model’s capability to fuse multi-scale information more effectively.Beside the ELA attention mechanism is incorporated into the in front of the structure to strengthen the representation of small targets and fine-grained details [24].
3. WIoUv3 is employed as the model’s loss function. By leveraging its dynamic non-monotonic focusing mechanism, it enables more accurate defect localization [25].

## Materials and methods

To address the issue of low detection accuracy in existing algorithms for wood board edge banding defects, this study proposes an improved detection algorithm named YOLOv8-MDEW. Specifically, a Multi-Channel Feature Fusion (MCFF) module is designed and integrated with the C2f structure to enhance multi-scale feature representation. Furthermore, an improved SPPF-D module is introduced based on the original SPPF, enabling the model to more effectively capture defect features at different scales. To further enhance feature extraction, the Efficient Local Attention (ELA) mechanism is incorporated. In addition, the original CIoU loss is replaced with WIoUv3 to accelerate convergence and improve localization precision. The overall architecture of YOLOv8-MDEW is illustrated in S1 Fig.

### C2f-MCFF

In YOLOv8, the C2f module serves as a critical component for feature extraction, employing convolutional and bottleneck structures to process intermediate representations. However, it relies on standard convolution operations, which constrain the receptive field and limit the model’s capacity to capture features from objects of varying scales. This limitation can result in incomplete feature representation, particularly for multi-scale or fine-grained targets, thereby reducing detection accuracy.

In the context of wood board edge defect detection, defects often exhibit irregular shapes, varying sizes, and appear against complex background textures. To address these challenges and enhance the model’s ability to extract discriminative features, we propose replacing the standard Bottleneck structure within the C2f module with a newly designed Multi-Channel Feature Fusion (MCFF) module, forming an enhanced structure termed C2f-MCFF.

The C2f-MCFF module incorporates the concept of grouped convolution to enrich feature diversity. Specifically, the input feature map is first passed through a 3×3 convolution layer, followed by batch normalization and SiLU activation. The output is then split into two branches, denoted as x1 and x2, each containing half of the original channels. The x1 branch is processed through a 3×3 convolutional, while x2 undergoes a 3×1 convolution to introduce asymmetric receptive fields. The outputs from x1, x2, and the initial convolution are subsequently concatenated, followed by a 1×1 convolution for channel adjustment and a residual connection for information preservation.

By integrating convolutions with varying kernel sizes and orientations, C2f-MCFF effectively enlarges the receptive field and enhances the extraction of features across multiple scales. This design is particularly beneficial for detecting slender and irregular edge defects on wood surfaces. Furthermore, the multi-branch structure improves feature fusion while maintaining critical defect-related information. The architecture of the improved C2f-MCFF module is illustrated in S2 and S3 Figs.

### SPPF-D

YOLOv8 incorporates the Spatial Pyramid Pooling – Fast (SPPF) module to expand the receptive field and facilitate global feature fusion. This module utilizes a spatial pyramid structure composed of convolutional operations followed by three consecutive max-pooling layers, aiming to capture multi-scale contextual information. However, relying solely on max pooling can result in the loss of fine-grained defect features, especially in scenarios where defect boundaries are subtle or partially occluded.

In the task of wood board edge defect detection, input images often contain noise, and defects such as gaps, chips, or residual glue typically exhibit small size and irregular morphology. Under these conditions, the original SPPF module may inadequately represent fine-scale defect features, potentially causing feature fusion loss and missed detections.

To address these limitations, we propose an enhanced module termed SPPF-D (SPPF with Dilation), which builds upon the original SPPF structure. The proposed SPPF-D introduces the Efficient Local Attention (ELA) mechanism to strengthen feature expressiveness, and incorporates dilated convolutions and average pooling to capture broader contextual dependencies while preserving spatial resolution. These additions aim to improve the module’s sensitivity to small and subtle defects, and mitigate feature loss during the pooling process.

The architectural structure of the SPPF-D module is illustrated in S4 Fig.

The input feature map is initially processed using a standard 1×1 convolution to adjust channel dimensions. Subsequently, a 3×3 dilated convolution is applied to expand the receptive field and enhance the representation of subtle and fine-grained features.

The resulting feature map is then propagated through two parallel branches. The first branch performs three consecutive max pooling operations to capture high-level semantic information and emphasize prominent features. In parallel, the second branch applies three consecutive average pooling operations to retain background context and low-frequency information.

The outputs from both branches are concatenated along the channel dimension to form a unified feature representation that integrates multi-scale contextual cues. A final 1×1 convolution is employed to fuse and compress the concatenated features, producing the output feature map.

The overall process can be mathematically formulated as follows:

### ELA Attention

In wood board edge defect detection, background clutter and wood grain textures often introduce irrelevant information, which may mislead the model and reduce detection accuracy. To address this challenge, an attention mechanism is introduced to enhance the model’s focus on salient features, thereby improving its overall detection performance. In this study, the Efficient Local Attention (ELA) module is integrated directly before the input of the SPPF-D module, serving as a lightweight spatial attention gate.

By placing ELA at the beginning of SPPF-D, the network is guided to emphasize informative regions in the feature map before multi-scale pooling operations. This design allows the downstream pooling layers to extract more discriminative spatial features, thereby enhancing overall feature fusion. Importantly, ELA achieves this without incurring significant computational overhead.

The core mechanism of ELA involves applying strided average pooling along the spatial dimensions (horizontal and vertical) for each channel, resulting in two feature descriptors that encode directional dependencies. This narrow kernel pooling strategy enables the module to capture long-range spatial correlations while suppressing background noise and irrelevant textures. The resulting horizontal and vertical descriptors are each passed through lightweight transformations to compute attention weights, which are then fused via element-wise multiplication to modulate the original features.

This process enables the model to focus more accurately on defect-related regions, improving localization precision while maintaining computational efficiency. The detailed architecture of the ELA module is illustrated in S5 Fig.

The input feature map is processed through a one-dimensional convolution, followed by group normalization and Sigmoid function to generate two positional attention weights *y*^*h*^ and *y*^*w*^. *y*^*h*^ is the horizontal positional attention weight, *y*^*w*^ is the vertical positional attention weight, σ is Sigmoid function, *F*_*h*_ and *F*_*w*_ is one-dimensional convolution, *G*_*n*_ is group normalization.The calculation formula is as follows Eq (1), Eq (2), Eq (3).


$$y^{h}=\sigma (G_{n}(F_{h}(z_{h})))$$ (1)

$$y^{w}=\sigma (G_{n}(F_{w}(z_{w})))$$ (2)

$$Y={\text{x}}_{c}\times y^{h}\times y^{w}$$ (3)

The final output can be expressed as the element-wise multiplication of the original input feature map with the two distinct positional attention weights *F*_*h*_ and *F*_*w*_. *x*_*c*_ is the original input feature map.

In wooden board edge defect detection, a single defect type may appear in diverse morphological forms, posing challenges for accurate identification. To address this, the Efficient Local Attention (ELA) mechanism is introduced to enhance the model’s ability to capture both spatial-semantic information and global contextual features. Owing to its lightweight design, ELA facilitates efficient extraction of defect-relevant features while enriching the overall feature representation. This ultimately contributes to improved detection accuracy, particularly for subtle or irregular edge defects.

Within the SPPF-D module, the input feature map is first refined by ELA to highlight informative regions. Subsequently, dilated convolutions are employed to enhance the representation of small-scale defects, followed by the fusion of multi-scale features through the combination of global max pooling and average pooling. This design enables more effective integration of contextual information across different spatial resolutions, thereby strengthening the model’s ability to detect and localize edge defects with complex distributions. As a result, the SPPF-D module significantly improves the robustness and reliability of detection under real-world conditions.

### WIoU

YOLOv8 adopts Complete IoU (CIoU) as its bounding box regression loss function, which considers factors such as the overlap area and the distance between the centers of the predicted and ground truth boxes. However, the aspect ratio penalty term in CIoU does not accurately capture the differences in width and height with respect to the actual confidence of the predicted boxes. This can lead to suboptimal optimization similarity, potentially hindering precise localization.

In real-world production environments, wood panel images are often collected under variable lighting conditions and equipment setups, leading to datasets that contain a substantial number of low-quality samples. In such scenarios, CIoU tends to place excessive emphasis on high-quality bounding boxes during regression. As a result, the model may become biased, reducing its generalization capability and limiting its robustness in detecting diverse defects under inconsistent imaging conditions.

To address the impact of variable-quality samples on bounding box regression, this study adopts WIoU v3 as the regression loss function. WIoU introduces a dynamic, non-monotonic focusing mechanism and replaces IoU with a novel outlier-aware score to evaluate anchor box quality. The outlier degree is calculated as shown in Eq (4):


$$\beta =\frac{{ℒ}_{IoU}^{*}}{{ℒ}_{IoU}},\beta \in [0,\infty )$$ (4)

A smaller outlier degree indicates higher-quality anchor boxes. To mitigate the influence of low-quality samples, WIoU assigns lower gradient gains to samples with higher outlier scores. Through this gradient gain allocation strategy, the harmful impact of low-quality samples is significantly reduced.

WIoU v3 constructs a non-monotonic focusing factor using the outlier score β to modulate the regression loss. Specifically, when the β=δ, *r* = 1, the gradient gain reaches its maximum. This ensures that high-quality samples receive more attention during optimization, while the effect of noisy or poorly labeled samples is down-weighted.

The WIoU loss is computed as shown in Eq (5), Eq (6), Eq (7):


$${ℒ}_{WIoUv1}=R_{WIoU}{ℒ}_{IoU}$$ (5)

$$R_{WIoU}=exp(\frac{(x-x_{gt}{)}^{2}+(y-y_{gt}{)}^{2}}{(W_{g}^{2}+H_{g}^{2}{)}^{*}})$$ (6)

$${ℒ}_{WIoUv3}=r{ℒ}_{WIoUv1},r=\frac{\beta }{\delta \alpha^{\beta -\delta }}$$ (7)

WIoU IoU as shown in S6 Fig, β is the outlier degree. ${ℒ}_{IoU}^{*}$ is the mean of the loss function. $\overset{―}{{ℒ}_{IoU}}$ is the moving average of momentum m. *R*_*WIoU*_ is attention mechanism. *x*, *y*, *x*_*gt*_, *y*_*gt*_ are the x and y coordinates of the center points of the predicted and ground-truth boxes. *W*_*g*_ and *H*_*g*_ are the width and height of the minimum enclosing bounding box. In order to prevent *R*_*WIoU*_ from generating gradients that hinder convergence. Here, the asterisk * denotes that the corresponding term is detached from the computational graph during backpropagation. That is, gradients are not propagated through *W*_*g*_ and *H*_*g*_. δ and α are hyperparameters that are empirically selected based on validation performance. They play a crucial role in the optimization process by dynamically re-weighting the gradient contributions, thereby focusing training on samples with more beneficial IoU characteristics. This mechanism improves the localization accuracy and facilitates better convergence behavior of the model.

In the task of wood panel edge defect detection, defect samples exhibit varying levels of quality and considerable variation in anchor box scales. By adopting WIoU as the regression loss function, the model’s convergence capability is significantly enhanced. This leads to more accurate localization of defects on the wood panel edges and effectively improves the overall detection performance of the model.

## Results and discussion

The images used in this experiment were collected from the panel production line of Xiamen GoldenHome Co. Ltd. The dataset was conducted using a self-built dataset comprising 6,000 images,including eight categories of wood edge banding defects. The annotations were conducted by a single trained annotator using the X-anylabeling tool. To ensure accuracy, ambiguous samples were discussed with on-site experts and resolved according to standardized defect definitions.As the dataset was labeled by only one individual, inter-annotator agreement metrics were not applicable.Each defect was annotated based on a consistent visual criterion to ensure clarity and reproducibility. The following provides a definition and annotation guideline for each category. Some defects are shown in S7 Fig. The experimental environment used in this study is shown in S1 Table.

1. **Short edge banding**: Missing or incompletely applied edge banding along the wood board edge. The exposed area of the board is labeled tightly to enclose only the uncovered region.
2. **Glue residue**: Overflow of adhesive beyond the edge banding boundary. Only glue residues exceeding approximately 1 mm beyond the band are annotated. Internal glue lines are excluded.
3. **Edge banding tackless**: Detachment or separation of the edge banding from the surface of the board. Regions where the band visibly lifts or peels off are labeled.
4. **Glue seam**: Incomplete bonding or unglued seams between the edge banding and the board. Clearly visible bonding gaps are annotated; minor cosmetic seams are ignored.
5. **Edge banding longer**: Edge banding that extends beyond the physical boundary of the wood board due to trimming failure. Only the protruding segments are labeled.
6. **Board gap**: Physical damage such as chipping, cracking, or breakage along the edge of the board. Only structural damage is annotated; superficial surface scratches are excluded.
7. **Edge banding dirty**: Visible dirt or stains on the surface of the edge banding. Label clearly visible, prominent dirt or stains; fine specks are ignored.
8. **Tape residue**: Foreign band-shapes objects or materials adhered to the edge banding surface.

The dataset is divided into train, valid, and test sets in a ratio of 8:1:1. The model training parameters are listed in S2 Table.

### Test indicators

To accurately evaluate the model’s performance in detecting wood board edge defects, this study adopts the following metrics: Precision (P), Recall (R), mean Average Precision (mAP50), and mAP50:95.

Precision (P) refers to the proportion of correctly predicted positive samples among all predicted positive samples. The calculation formula for precision is as shown in Eq (8):


$$P=\frac{TP}{TP+FP}$$ (8)

Recall (R) refers to the proportion of actual positive samples that are correctly predicted as positive. The calculation formula for recall is as shown in Eq (9):


$$R=\frac{TP}{TP+FN}$$ (9)

In the above formulas,TP is the number of correctly predicted positive samples,FN is the number of actual positive samples incorrectly predicted as negative,FP is the number of negative samples incorrectly predicted as positive. mAP (mean Average Precision) is one of the most commonly used evaluation metrics in object detection. It takes into account the detection performance across different classes and consolidates them into a single evaluation metric.In object detection, a higher mAP value indicates better model performance. The general formula is shown in Eq (10), Eq (11):


$$AP=\sum_{i=1}^{n-1}(r_{i+1}-r_{i})p(r_{i+1})$$ (10)

$$mAP=\frac{1}{m}\sum_{i=1}^{m}AP_{i}$$ (11)

### Attention mechanism comparison experiments

To validate the effectiveness of the ELA attention mechanism within the proposed algorithm, we conducted comparative experiments using several attention mechanisms, including CAFM, CBAM, SimAM, and EMA. The experimental results are summarized in S3 Table.

As shown in S3 Table the incorporation of the ELA attention mechanism results in the most substantial and consistent improvement across all evaluation metrics. ELA achieves the highest mAP50 (0.732) and competitive mAP50:95 (0.394), while also maintaining a favorable balance between precision and recall. These results indicate that ELA effectively enhances the model’s ability to detect edge defects on wood panels.

Although the EMA module records a slightly higher mAP50:95 (0.395), it underperforms compared to ELA in terms of both mAP50 and recall, suggesting weaker overall detection capability. Similarly, the SimAM module yields a marginal improvement in Recall (by 0.4%), but this comes at the cost of a 0.2% drop in mAP50 and a 1.3% decrease in Precision, reflecting a trade-off that may not be ideal for the task at hand.

The CAFM module demonstrates a relatively higher recall (0.678), but exhibits a notable decline in both mAP50 (0.728) and mAP50:95 (0.391), indicating unstable regression performance. CBAM shows moderate results overall but does not surpass ELA in whole individual metric.

These findings highlight that among the attention mechanisms evaluated, ELA offers the most balanced enhancement to detection accuracy and consistency. Its superior performance validates its integration into the proposed YOLO-MDEW architecture for industrial edge defect detection applications.

### Comparative study of loss functions

YOLOv8 employs the Complete IoU (CIoU) loss function, which has been observed to exhibit relatively slow convergence and suboptimal bounding box regression performance when applied to edge defect detection tasks. To evaluate the effectiveness of Wise-IoU (WIoU) in improving bounding box regression for such fine-grained defects, a comparative study was conducted against several mainstream IoU-based loss functions. The results are presented in S4 Table.

The results demonstrate that WIoU consistently outperforms CIoU and other loss functions across all evaluation metrics. Compared to CIoU, WIoU improves precision, recall, mAP50, and mAP50:95 by 0.9%, 0.8%, 0.6%, and 0.4%, respectively. While other IoU Loss falls behind WIoU in terms of both mAP metrics. These findings indicate that WIoU provides a more balanced enhancement in both localization accuracy and classification stability.

To further investigate the impact of WIoU’s hyperparameters—α and β—on performance, a series of experiments were conducted with different combinations. The results are shown in S5 Table.

Among the tested configurations, the combination of α=1.5 and β=2.6 yielded the best overall performance, achieving the highest mAP50 and mAP50:95 values. Deviations from this setting resulted in slight declines in performance, indicating that the values of α and β play a crucial role in controlling the non-monotonic focusing factor of the WIoU loss and, consequently, the gradient distribution during training. In summary, WIoU demonstrates superior capability in reducing bounding box regression errors and improving overall detection accuracy when compared with existing IoU-based losses. Additionally, appropriate tuning of its hyperparameters further enhances its effectiveness, underscoring its suitability for complex defect detection tasks in industrial scenarios.

### Ablation experiments

To evaluate the effectiveness of each proposed module, ablation experiments were conducted and the results are presented in S6 Table. The baseline model (Model 1) corresponds to the original YOLOv8n, which achieves an mAP50 of 0.721 ± 0.007 and mAP50:95 of 0.390 ± 0.002 with 2.7M parameters and 6.9 GFLOPs.

In Model 2, the integration of the SPPF-D module leads to a performance gain, increasing mAP50 by 1.1% and mAP50:95 by 0.4%, while maintaining computational efficiency (2.9M parameters and 7.1 GFLOPs). This confirms that the SPPF-D structure effectively enhances multi-scale feature fusion and improves defect localization.

Model 3 builds upon Model 2 by incorporating the C2f-MCFF module. This addition brings further improvements, with mAP50 reaching 0.734 ± 0.007 and mAP50:95 rising to 0.396 ± 0.003. Compared to the baseline, this represents an overall gain of 0.2% in mAP50 and 0.2% in mAP50:95, demonstrating that MCFF enhances the model’s capacity to extract discriminative features.

Model 4 introduces the Wise-IoU (WIoU) loss function on top of Model 3. While mAP50 slightly increases to 0.740 ± 0.003, mAP50:95 also improves to 0.400 ± 0.003. Importantly, the inclusion of WIoU results in a noticeable refinement in localization performance, as indicated by the consistent gains across all metrics. The model complexity remains nearly unchanged compared to Model 3, with 3.2M parameters and 7.5 GFLOPs.

### Comparative experiments

To further validate the performance of the proposed improved algorithm, comparative experiments were conducted between the proposed method and the latest YOLO series algorithms on a custom-built dataset. The training parameters and experimental settings were kept consistent with the initial configuration. The experimental results are presented in S7 Table

The results clearly demonstrate that YOLO-MDEW achieves the highest detection accuracy among all compared models. Specifically, it attains a mean mAP50 of 0.740 and mAP50:95 of 0.400, outperforming YOLOv8n by 1.9% and YOLOv9t, YOLOv10n, YOLOv11n, and YOLOv12n by 2.3%, 5.2%, 2.7%, and 2.7%, respectively, in terms of mAP50. The mAP50:95 also shows consistent improvement, with increases of 1.0%, 1.9%, 3.1%, 1.4%, and 2.3% over the respective baselines.

In addition to overall accuracy, YOLO-MDEW achieves the highest mean precision (0.756) and competitive recall (0.668), with relatively low standard deviations across multiple runs, indicating both strong performance and training stability. Compared to YOLOv8n, the precision and recall improve by 3.5% and 0.2%, respectively. Consistent gains are also observed over other YOLO-based baselines, including YOLOv10n, where improvements of 5.0% in precision and 4.6% in recall are achieved.

Although YOLO-MDEW introduces a slightly higher computational cost (3.2M parameters and 7.5 GFLOPs) compared to YOLOv8n (2.7M parameters and 6.9 GFLOPs), the increase remains marginal relative to the performance gains. In terms of inference efficiency, the proposed model achieves 119 FPS, which, although slightly lower than YOLOv8n (131 FPS) and YOLOv10n (133 FPS), is still far beyond the real-time requirement for industrial inspection (2.5–3 FPS), ensuring practical deployment feasibility.

To further evaluate the generalization capability of the proposed method beyond YOLO-based detectors, a representative transformer-based model (RT-DETR) is included for comparison. As shown in S8 Table, RT-DETR achieves competitive performance (mAP50 of 0.723 and mAP50:95 of 0.395), but at a significantly higher computational cost and lower inference speed (28 FPS). In contrast, YOLO-MDEW achieves better accuracy while maintaining substantially higher efficiency, making it more suitable for real-time industrial applications.

The mAP50 curves of YOLO-MDEW and YOLOv8n are presented in S8 Fig to illustrate training dynamics. The proposed method exhibits more stable convergence behavior, indicating improved optimization efficiency.

Overall, the experimental results demonstrate that YOLO-MDEW achieves a balanced improvement in accuracy, efficiency, and stability. These characteristics make it well-suited for practical industrial defect detection scenarios.

### Scale-wise performance analysis

To further evaluate the effectiveness of the proposed method in handling objects of different sizes, scale-specific recall metrics are analyzed for small, medium, and large defect instances. As shown in S9 Table, YOLO-MDEW consistently outperforms the baseline YOLOv8n across all object scales. Specifically, recall improves from 65.6% to 67.8% for small objects, from 74.1% to 76.8% for medium objects, and from 67.3% to 72.9% for large objects.

Notably, the most significant improvement is observed in large-scale defects, with a gain of 5.6%, suggesting that the proposed feature fusion strategy effectively enhances the model’s ability to capture more comprehensive contextual information. Meanwhile, the consistent improvements in small and medium objects indicate that the model maintains stable sensitivity to fine-grained and intermediate-scale features.

These results demonstrate that YOLO-MDEW achieves consistent performance improvements across different object scales, providing strong quantitative evidence for the effectiveness of the proposed multi-scale feature enhancement design.

### Confusion matrix comparison

Beyond scale-wise performance, a more detailed class-level analysis is conducted using confusion matrices. As shown in S9 Fig, the confusion matrices for YOLOv8 (left) and the proposed YOLO-MDEW model (right) reveal clear differences in classification behavior. In a confusion matrix, higher values along the diagonal indicate greater classification accuracy. Compared to YOLOv8, the YOLO-MDEW model demonstrates an overall improvement in defect classification accuracy. Notably, the accuracy for the Edge banding tackless category increases by 6%, and Edge banding longer improves by 3%. Except for a slight decline in the Board gap category, classification performance across the remaining categories is either comparable or improved. Additionally, YOLO-MDEW reduces confusion between similar defects: while YOLOv8 exhibits a 1% misclassification rate for Board gap, Tape residual, and Edge banding dirty,the improved model further reduces these errors, indicating enhanced reliability and robustness in edge defect recognition.

To further validate these improvements, we report per-class Precision, Recall, and mAP50 for both models in S10 and S11 Tables. YOLO-MDEW achieves notable gains across most defect categories. For example, in the Glue seam category, Precision and Recall increase by 1.7% and 1.1%, respectively, with a 4.5% gain in mAP50. In the Edge banding longer category, improvements of 1.9% in Precision and 3.9% in Recall are observed. Minor drops in Precision are seen in categories like Tape residue and Short edge banding, though these are often offset by increases in Recall. One notable exception is the Board gap category, where Recall decreases by 1.3% and mAP50 decreases by 1.9% despite an increase in Precision.

Further analysis suggests that this performance degradation is likely attributed to the inherent difficulty of the Board gap category. Specifically, some instances exhibit extremely small and weakly distinguishable visual cues, which can be easily confused with normal edges under complex lighting and textured backgrounds. In addition, Board gap defects often co-occur with other edge-related imperfections such as glue residue and seam cracks, leading to inter-class similarity and spatial ambiguity.

Meanwhile, a secondary contributing factor may stem from the multi-scale feature learning process. Although the proposed C2f-MCFF and SPPF-D modules enhance feature representation across scales, although overall performance is consistently improved across all scales, minor variations may still appear under different evaluation conditions due to IoU-based matching sensitivity due to optimization trade-offs and IoU-based matching sensitivity during evaluation.

Overall, these factors jointly explain the minor performance variation in the Board gap category, while the overall robustness of YOLO-MDEW remains unaffected.

### Comparison of detection results

To intuitively demonstrate the performance difference between YOLOv8 and the proposed YOLO-MDEW model in edge defect detection for wooden boards, we compared the detection results of both models across various defect types, as illustrated in S10 Fig. On the left is shown the detection output of YOLOv8n, while right presents the results from YOLOv8-MDEW.

In the first three sets of images, YOLOv8n exhibits notable instances of missed detections. Specifically, in the third set, where multiple defect types co-exist, YOLOv8n fails to detect the glue line at the bottom of the image. In contrast, YOLOv8-MDEW successfully identifies all present defects, including glue line, edge chipping, and residual tape.

In the following four image sets, YOLOv8n struggles to accurately detect continuous defects of the same type, often resulting in incomplete localization. YOLOv8-MDEW, on the other hand, demonstrates a superior ability to capture the full extent of such defects with higher accuracy.In the final two image sets, YOLOv8n produces several false positives and incorrect classifications, whereas YOLOv8-MDEW maintains better precision and reliability.

Overall, the experimental results clearly indicate that the proposed YOLOv8-MDEW model outperforms the baseline YOLOv8n in terms of detection accuracy, robustness, and its ability to recognize and localize diverse defect types on wooden board edges, thereby demonstrating its practical value in industrial defect inspection scenarios.

## Conclusion

This paper proposes an improved YOLOv8-MDEW algorithm for wood panel edge defect detection. First, an MCFF module is designed and integrated into a novel C2f-MCFF structure to enhance the model’s multi-scale feature extraction capability. Then, an improved SPPF-D module is introduced to strengthen feature fusion and preserve more effective information. Additionally, the ELA attention mechanism is incorporated to enhance spatial semantic representation and improve the characterization of defect features. Finally, the CIoU loss is replaced with WIoU to accelerate convergence and improve localization accuracy.

Experimental results demonstrate that YOLOv8-MDEW achieves consistent performance improvements, with gains of 1.9% and 1.0% in mAP50 and mAP50:95, respectively. The model also shows improved capability in distinguishing different defect types. Overall, the proposed method outperforms baseline and comparative models, confirming the effectiveness of the introduced improvements.

Despite these promising results, this study has certain limitations. First, the evaluation is conducted on a custom dataset focusing on specific edge defects, which may limit the generalizability to other defect types or industrial scenarios. Second, although real-time performance is maintained, the increased model complexity may pose challenges for deployment on resource-constrained devices.

In future work, we will explore lightweight model designs to further reduce computational cost and enable efficient deployment on edge devices. In addition, more diverse defect samples will be collected from wood panels made of different materials, such as medium-density fiberboard (MDF), particleboard, and plywood, to improve generalization capability.

Furthermore, domain adaptation strategies will be investigated to enhance cross-domain robustness. For example, adapting models trained on controlled laboratory datasets to real-world production environments with varying lighting conditions, surface textures, and imaging devices. Future efforts may focus on feature distribution alignment across different production lines and transferring knowledge from wood defect datasets to other surface inspection tasks, such as metal surface defects and laminated materials.

These directions are expected to further improve the generalization ability and practical applicability of the proposed method in real industrial inspection systems.

## Supporting information

S1 Fig The structure of the YOLO-MDEW. (TIF)

S2 Fig The structure of the Bottleneck-MCFF Module. (TIF)

S3 Fig The structure of the C2f-MCFF Module. (TIF)

S4 Fig The structure of the SPPF-D. (TIF)

S5 Fig ELA Attention Mechanism. (TIF)

S6 Fig WIoU Loss IoU. (TIF)

S7 Fig Board Band Edge Defect. (TIF)

S8 Fig The mAP50 curve of training process. (TIF)

S9 Fig The confusion matrix. (TIF)

S10 Fig Comparison of different defect detection results. (TIF)

S1 Table Environment configuration. (DOCX)

S2 Table Hyperparameters configuration. (DOCX)

S3 Table Attention mechanism comparison experiments. (DOCX)

S4 Table Comparative Study of Loss Functions. (DOCX)

S5 Table Hyperparameter experiments. (DOCX)

S6 Table Results of the ablation experiments. (DOCX)

S7 Table Comparison of different models. (DOCX)

S8 Table Comparison between RT-DETR and YOLO-MDEW. (DOCX)

S9 Table Scale-wise Recall for YOLOv8 and YOLO-MDEW. (DOCX)

S10 Table Per-class Precision and Recall for YOLOv8. (DOCX)

S11 Table Per-class Precision and Recall for YOLO-MDEW. (DOCX)

## References

[1] Lu J. China furniture industry economic operation briefing released. China Consumer Daily. 2025:A02. doi: 10.28866/n.cnki.nxfrb.2025.000229

[2] Zhou Y. Research on quality management of manufacturing process in panel customized furniture enterprises. Changsha: Central South University of Forestry and Technology; 2018.

[3] Su H, Zhang J, Zhang B. A review of surface defect detection based on visual perception. Comput Integr Manuf Syst. 2023;29(1):169–91. doi: 10.13196/j.cims.2023.01.015

[4] Çınar H. Eco-design and furniture: environmental impacts of wood-based panels, surface and edge finishes. For Prod J. 2005;55(11):27–33.

[5] Qin H, Chen X, Zhang Y. Environmental chamber test and modeling study on factors affecting formaldehyde emission from particleboard. Chin J Environ Sci. 2009;29(8):801–5.

[6] Chen X, Wu Z. Analysis of methods to improve the edge banding quality and efficiency of panel furniture based on intelligent manufacturing. J Furniture. 2020;41(3):75–8, 109. doi: 10.16610/j.cnki.jiaju.2020.03.017

[7] Liu W, Anguelov D, Erhan D, Szegedy C, Reed S, Fu C-Y, et al. SSD: single shot multibox detector. In: Lecture notes in computer science. Springer International Publishing; 2016. 21–37. 10.1007/978-3-319-46448-0_2

[8] Redmon J, Divvala S, Girshick R, Farhadi A. You only look once: unified, real-time object detection. In: 2016 IEEE Conference on Computer Vision and Pattern Recognition (CVPR), 2016. 779–88. 10.1109/cvpr.2016.91

[9] Redmon J, Farhadi A. YOLOv3: an incremental improvement. arXiv. 2018. https://arxiv.org/abs/1804.02767

[10] Li C, Li L, Jiang H. YOLOv6: a single-stage object detection framework for industrial applications. arXiv. 2022. https://arxiv.org/abs/2209.02976

[11] Lin T-Y, Goyal P, Girshick R, He K, Dollar P. Focal loss for dense object detection. In: 2017 IEEE International Conference on Computer Vision (ICCV), 2017. 2999–3007. 10.1109/iccv.2017.324

[12] Girshick R, Donahue J, Darrell T, Malik J. Rich feature hierarchies for accurate object detection and semantic segmentation. In: 2014 IEEE conference on computer vision and pattern recognition, 2014. 580–7. 10.1109/cvpr.2014.81

[13] Xie S, Girshick R, Dollar P, Tu Z, He K. Aggregated Residual Transformations for Deep Neural Networks. In: 2017 IEEE Conference on Computer Vision and Pattern Recognition (CVPR), 2017. 5987–95. 10.1109/cvpr.2017.634

[14] Huang G, Liu Z, Van Der Maaten L, Weinberger KQ. Densely connected convolutional networks. In: 2017 IEEE conference on computer vision and pattern recognition (CVPR), 2017. 2261–9. 10.1109/cvpr.2017.243

[15] Zhang X, Zhai D, Chen J. Research on wood defect image reconstruction and quality evaluation model based on deep reinforcement learning. Hubei Agric Sci. 2020;59(13):140–5. doi: 10.14088/j.cnki.issn0439-8114.2020.13.032

[16] Cui W, Li Z, Duanmu A, Xue S, Guo Y, Ni C, et al. CCG-YOLOv7: a wood defect detection model for small targets using improved YOLOv7. IEEE Access. 2024;12:10575–85. doi: 10.1109/access.2024.3352445

[17] Zhou S, Zhu H, Liu X. Wood surface defect detection based on improved YOLOv8s. IAENG Inter J Comp Sci. 2024;51:186–94.

[18] Qiang Y, Wang X, Liu X, Wang Y, Zhang W. Edge-enhanced graph attention network for driving decision-making of autonomous vehicles via deep reinforcement learning. Proceed Instit Mech Eng Part D: J Automobile Eng. 2024;239(4):1168–80. doi: 10.1177/09544070231217762

[19] Sun H, Liu X, Huang B, Li W, Fang Y, Wang Y. Improved mode decomposition method for vibration signal denoising of aero-engine shaft bearing. Mechanical Systems and Signal Processing. 2025;238:113201. doi: 10.1016/j.ymssp.2025.113201

[20] Song Z, Liu X, Fang Y, Wang X, Su S. Topology optimization for cold plate using neural networks as proxy models. Eng Optimizat. 2024;56(12):2359–86. doi: 10.1080/0305215x.2024.2308555

[21] Chen L-C, Pardeshi MS, Lo W-T, Sheu R-K, Pai K-C, Chen C-Y, et al. Edge-glued wooden panel defect detection using deep learning. Wood Sci Technol. 2022;56(2):477–507. doi: 10.1007/s00226-021-01316-3

[22] Du X. Edge defect detection of wood panel based on improved YOLOv5. Chengdu: Xihua University; 2023.

[23] He H. Edge defect detection of wood panel based on improved YOLOv5. Beijing: University of Chinese Academy of Sciences; 2024.

[24] Xu W, Wan Y. ELA: efficient local attention for deep convolutional neural networks [preprint]. arXiv; 2024. doi:10.48550/arXiv.2403.01123

[25] Tong ZJ, Chen YH, Xu ZW. Wise-IoU: bounding box regression loss with dynamic focusing mechanism. arXiv. 2023. doi: 10.48550/arXiv.2301.10051

