# Supplementary figures and images for: YOLO-MDEW:Improved YOLOv8 for application of wood board edge banding defect detection
Source: PLoS One. 2026 May 8;21(5):e0348758. doi: 10.1371/journal.pone.0348758 (PMC13155551; doi:10.1371/journal.pone.0348758)

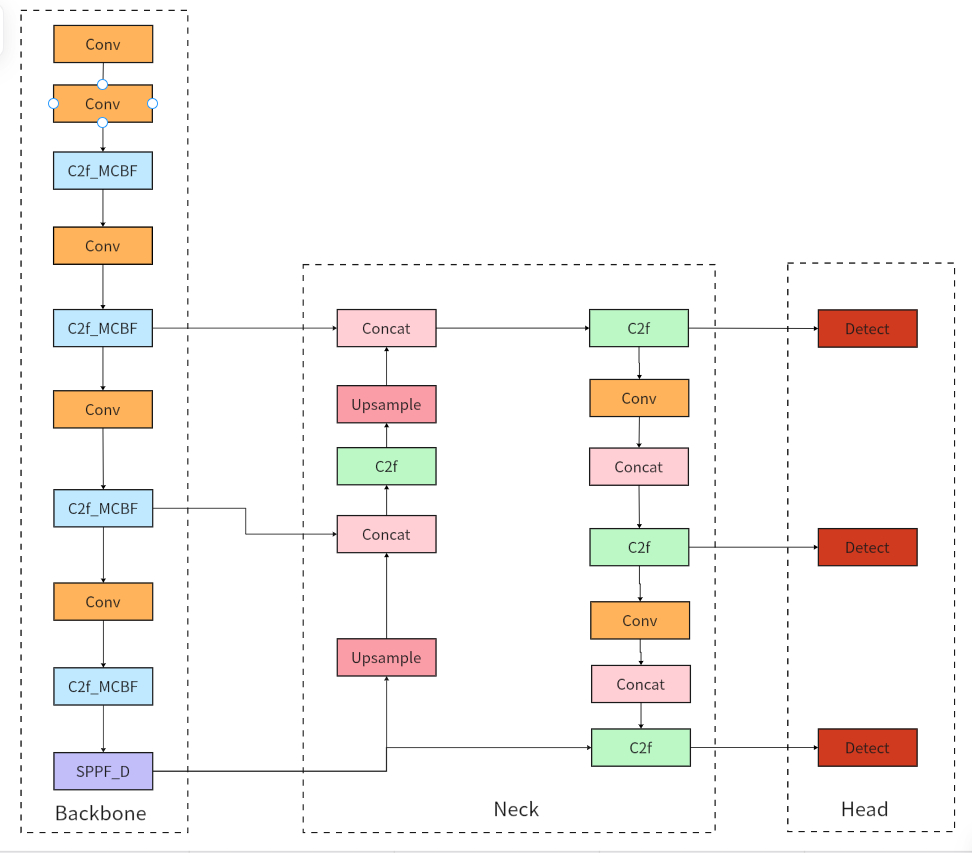

Supplement: S1 Fig — (TIF) [file pone.0348758.s001.tif]

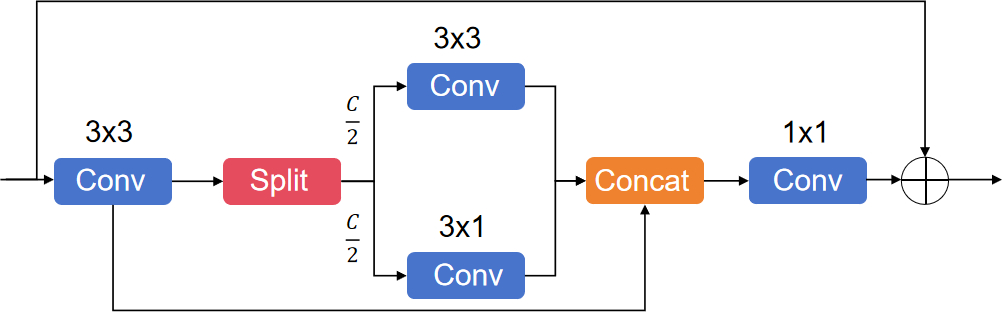

Supplement: S2 Fig — (TIF) [file pone.0348758.s002.tif]

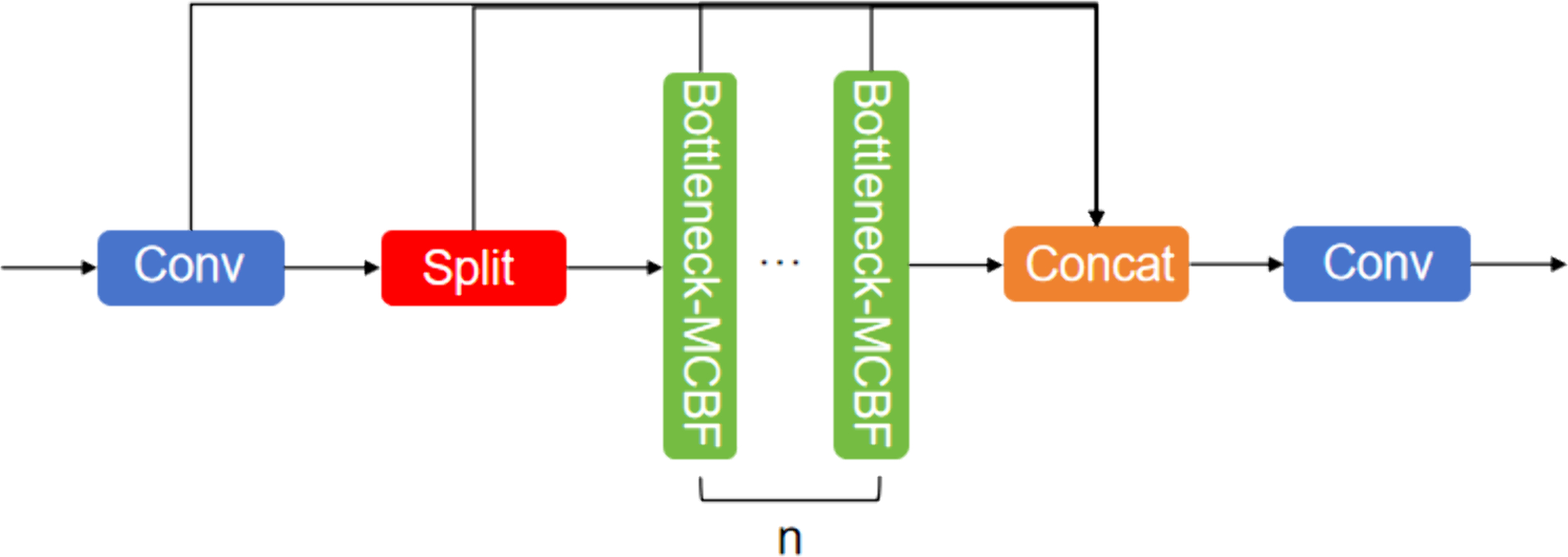

Supplement: S3 Fig — (TIF) [file pone.0348758.s003.tif]

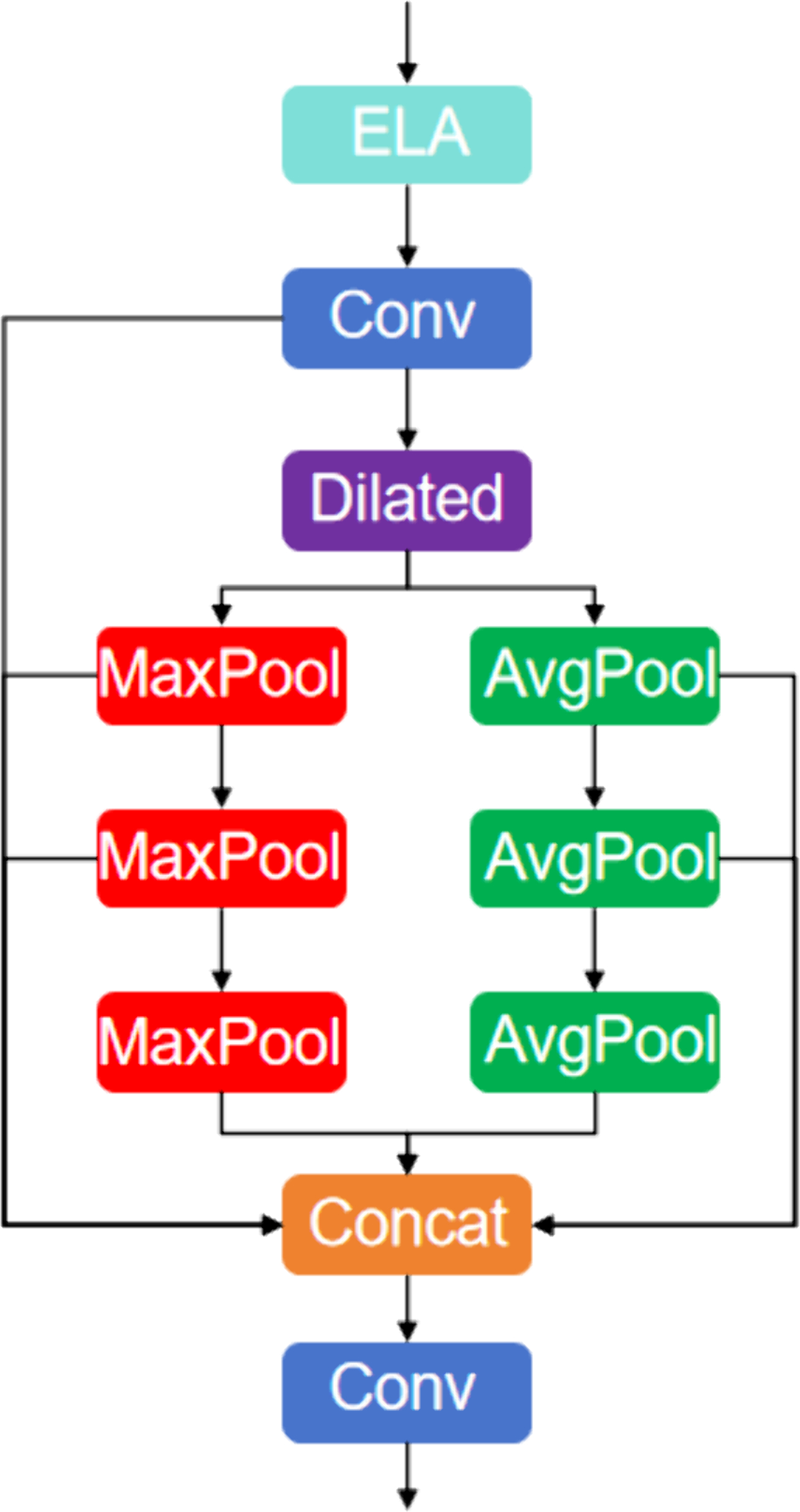

Supplement: S4 Fig — (TIF) [file pone.0348758.s004.tif]

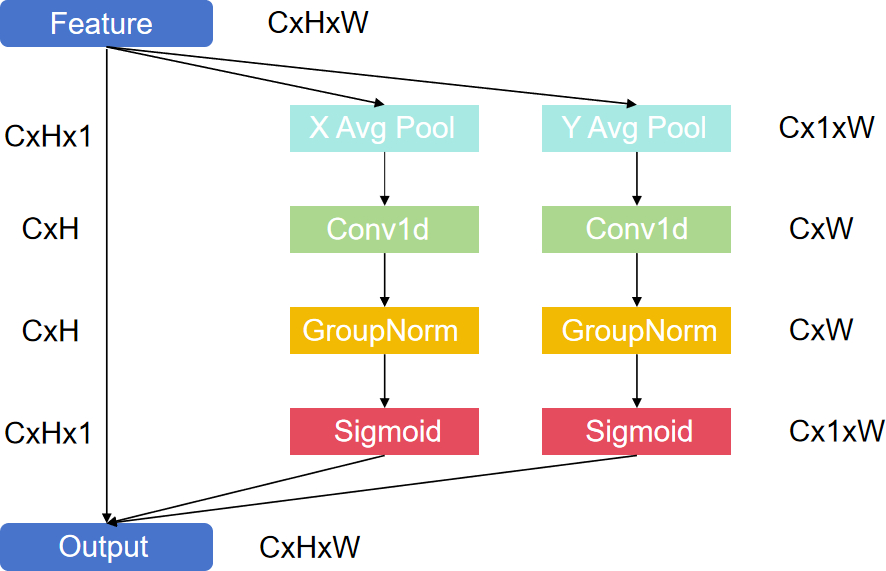

Supplement: S5 Fig — (TIF) [file pone.0348758.s005.tif]

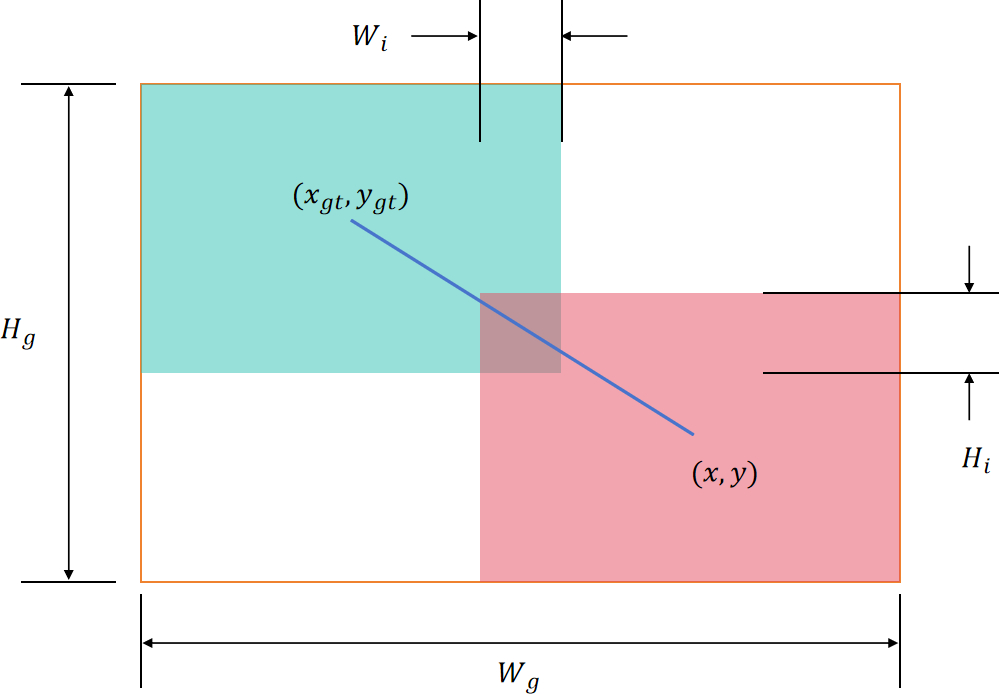

Supplement: S6 Fig — (TIF) [file pone.0348758.s006.tif]

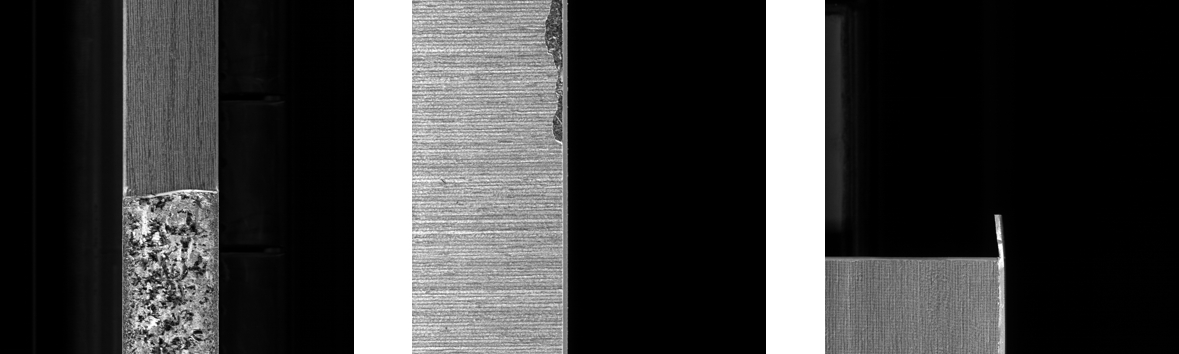

Supplement: S7 Fig — (TIF) [file pone.0348758.s007.tif]

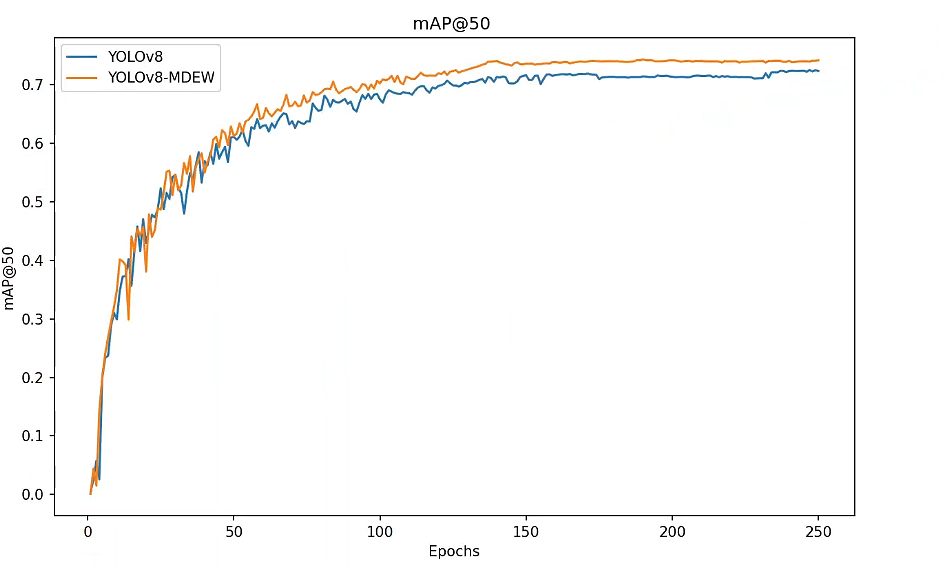

Supplement: S8 Fig — (TIF) [file pone.0348758.s008.tif]

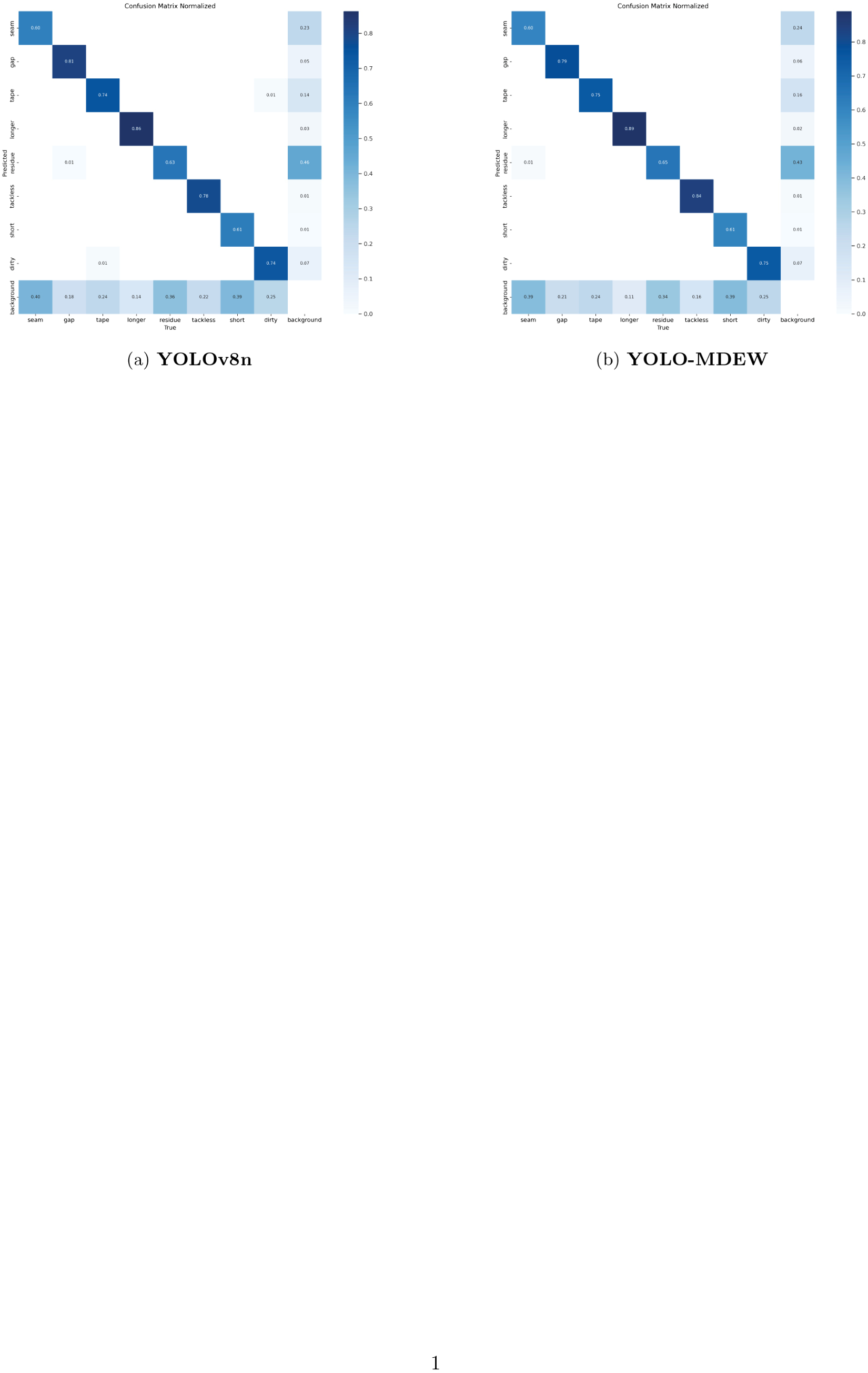

Supplement: S9 Fig — (TIF) [file pone.0348758.s009.tif]

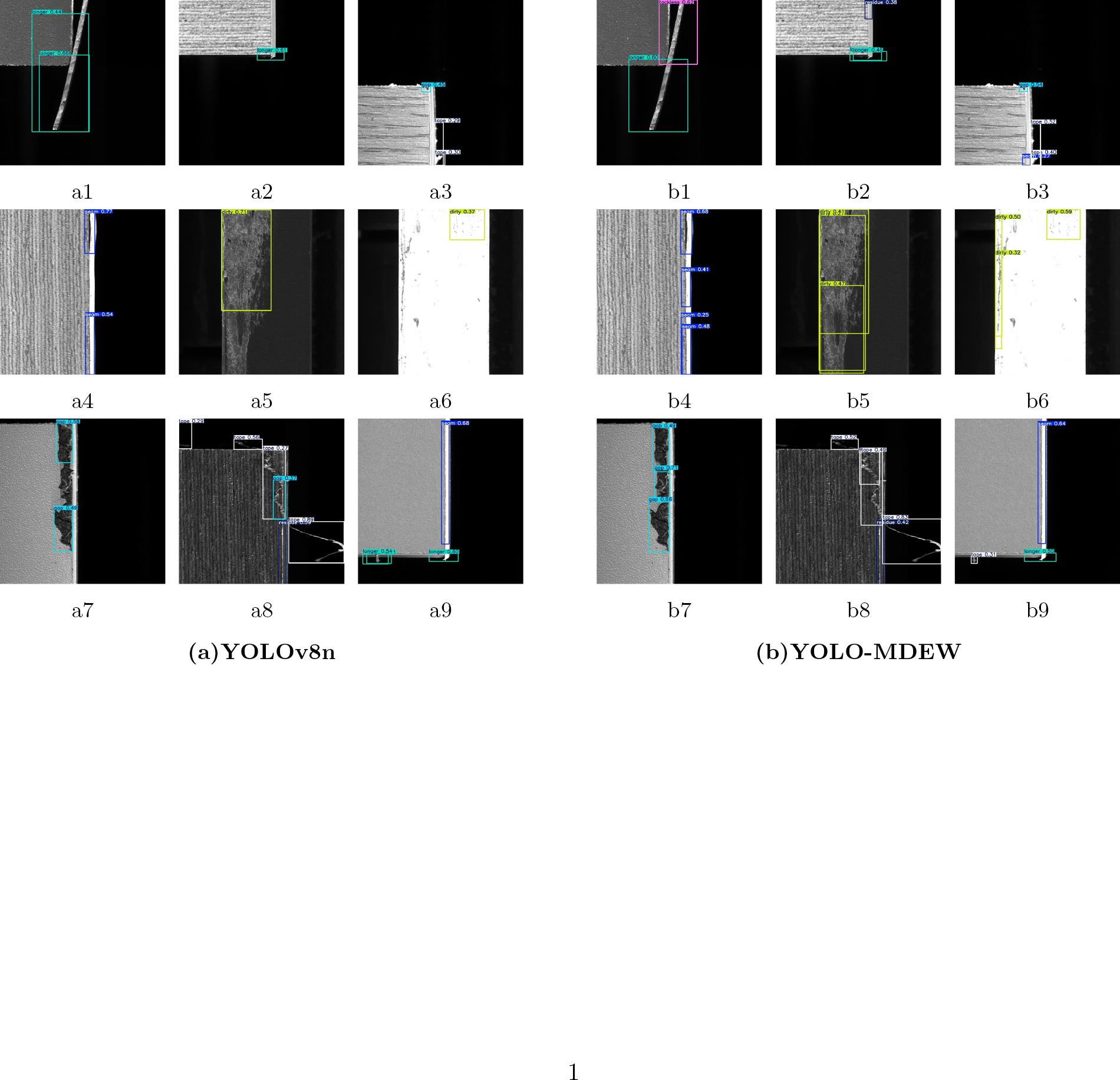

Supplement: S10 Fig — (TIF) [file pone.0348758.s010.tif]
