# Supplementary material for: YOLO-MDEW:Improved YOLOv8 for application of wood board edge banding defect detection
Source: PLoS One. 2026 May 8;21(5):e0348758. doi: 10.1371/journal.pone.0348758 (PMC13155551; doi:10.1371/journal.pone.0348758)
Supplement: S1 Table — (DOCX) [file pone.0348758.s011.docx]

S1 Table. Environment configuration.

|  | **Item** | **Configuration** |
| --- | --- | --- |
|  | Operating System | Linux |
|  | GPU | RTX 4090 |
|  | CPU | Xeon(R) Platinum 8352V |
|  | Python | 3.12 |
|  | Pytorch | 2.4.1 |
|  | Cuda | 12.6 |
