# Supplementary material for: YOLO-MDEW:Improved YOLOv8 for application of wood board edge banding defect detection
Source: PLoS One. 2026 May 8;21(5):e0348758. doi: 10.1371/journal.pone.0348758 (PMC13155551; doi:10.1371/journal.pone.0348758)
Supplement: S2 Table — (DOCX) [file pone.0348758.s012.docx]

S2 Table. Hyperparameters configuration.

|  | **Hyperparameters** | **Value** |
| --- | --- | --- |
|  | Lr0 | 0.01 |
|  | Image Size | 640×640 |
|  | Workers | 8 |
|  | Weight Decay | 0.0005 |
|  | Batch Size | 32 |
|  | Epoch | 250 |
