# Supplementary material for: YOLO-MDEW:Improved YOLOv8 for application of wood board edge banding defect detection
Source: PLoS One. 2026 May 8;21(5):e0348758. doi: 10.1371/journal.pone.0348758 (PMC13155551; doi:10.1371/journal.pone.0348758)
Supplement: S3 Table — (DOCX) [file pone.0348758.s013.docx]

S3 Table. Attention mechanism comparison experiments.

|  | **Attention** | **P** | **R** | **mAP50** | **mAP50:95** |
| --- | --- | --- | --- | --- | --- |
|  | ELA | 0.747±0.013 | 0.665±0.007 | 0.732±0.003 | 0.394±0.004 |
|  | SimAM | 0.734±0.012 | 0.669±0.001 | 0.730±0.005 | 0.394±0.004 |
|  | CAFM | 0.718±0.028 | 0.678±0.013 | 0.728±0.005 | 0.391±0.002 |
|  | EMA | 0.729±0.013 | 0.654±0.011 | 0.724±0.004 | 0.395±0.008 |
|  | CBAM | 0.744±0.018 | 0.659±0.016 | 0.728±0.010 | 0.394±0.002 |
