# Supplementary material for: YOLO-MDEW:Improved YOLOv8 for application of wood board edge banding defect detection
Source: PLoS One. 2026 May 8;21(5):e0348758. doi: 10.1371/journal.pone.0348758 (PMC13155551; doi:10.1371/journal.pone.0348758)
Supplement: S4 Table — (DOCX) [file pone.0348758.s014.docx]

S4 Table. Comparative study of loss functions.

|  | **IoU Loss** | **P** | **R** | **mAP50** | **mAP50:95** |
| --- | --- | --- | --- | --- | --- |
|  | CIoU | 0.747±0.022 | 0.66±0.008 | 0.734±0.007 | 0.396±0.003 |
|  | WIoU | 0.756±0.024 | 0.668±0.016 | 0.740±0.003 | 0.400±0.003 |
|  | EIoU | 0.733±0.018 | 0.620±0.009 | 0.700±0.002 | 0.386±0.001 |
|  | MPDIoU | 0.731±0.025 | 0.652±0.003 | 0.720±0.005 | 0.389±0.005 |
|  | DIoU | 0.736±0.003 | 0.658±0.003 | 0.716±0.003 | 0.385±0.003 |
|  | SIoU | 0.733±0.015 | 0.648±0.010 | 0.716±0.001 | 0.385±0.001 |
