# Supplementary material for: YOLO-MDEW:Improved YOLOv8 for application of wood board edge banding defect detection
Source: PLoS One. 2026 May 8;21(5):e0348758. doi: 10.1371/journal.pone.0348758 (PMC13155551; doi:10.1371/journal.pone.0348758)
Supplement: S5 Table — (DOCX) [file pone.0348758.s015.docx]

S5 Table. Hyperparameter experiments.

|  | $\alpha$ | $\beta$ | **mAP50** | **mAP50:95** |
| --- | --- | --- | --- | --- |
|  | 1.5 | 2.5 | 0.736±0.003 | 0.395±0.002 |
|  | 1.5 | 2.6 | 0.740±0.003 | 0.400±0.003 |
|  | 1.4 | 2.6 | 0.724±0.003 | 0.389±0.003 |
|  | 1.6 | 2.8 | 0.730±0.003 | 0.398±0.001 |
|  | 1.7 | 2.8 | 0.731±0.008 | 0.394±0.005 |
