# Supplementary material for: YOLO-MDEW:Improved YOLOv8 for application of wood board edge banding defect detection
Source: PLoS One. 2026 May 8;21(5):e0348758. doi: 10.1371/journal.pone.0348758 (PMC13155551; doi:10.1371/journal.pone.0348758)
Supplement: S6 Table — (DOCX) [file pone.0348758.s016.docx]

S6 Table. Results of the ablation experiments.

|  | **Models** | **SPPF-D** | **MCFF** | **WIoU** | **mAP50** | **mAP50:95** | **Parameters/M** | **GFLOPS** |
| --- | --- | --- | --- | --- | --- | --- | --- | --- |
|  | 1 |  |  |  | 0.721±0.007 | 0.390±0.002 | 2.7 | 6.9 |
|  | 2 | √ |  |  | 0.732±0.003 | 0.394±0.004 | 2.9 | 7.1 |
|  | 3 | √ | √ |  | 0.734±0.007 | 0.396±0.003 | 3.2 | 7.4 |
|  | 4 | √ | √ | √ | 0.740±0.003 | 0.400±0.003 | 3.2 | 7.5 |
