# Supplementary material for: YOLO-MDEW:Improved YOLOv8 for application of wood board edge banding defect detection
Source: PLoS One. 2026 May 8;21(5):e0348758. doi: 10.1371/journal.pone.0348758 (PMC13155551; doi:10.1371/journal.pone.0348758)
Supplement: S7 Table — (DOCX) [file pone.0348758.s017.docx]

S7 Table. Comparison of different models.

|  | **Models** | **P** | **R** | **mAP50** | **mAP50:95** | **FPS** | **Parameters/M** | **GFLOPS** |
| --- | --- | --- | --- | --- | --- | --- | --- | --- |
|  | YOLOv8n | 0.721±0.009 | 0.666±0.009 | 0.721±0.007 | 0.390±0.002 | 131 | 2.7 | 6.9 |
|  | YOLOv9t | 0.714±0.022 | 0.654±0.004 | 0.717±0.007 | 0.381±0.005 | 93 | 1.8 | 6.7 |
|  | YOLOv10n | 0.706±0.004 | 0.622±0.009 | 0.688±0.01 | 0.369±0.008 | 133 | 2.7 | 8.4 |
|  | YOLOv11n | 0.695±0.008 | 0.66±0.006 | 0.713±0.004 | 0.386±0.01 | 114 | 2.6 | 6.4 |
|  | YOLOv12n | 0.703±0.017 | 0.669±0.01 | 0.713±0.002 | 0.377±0.005 | 109 | 2.4 | 6.3 |
|  | OURS | 0.756±0.024 | 0.668±0.016 | 0.740±0.003 | 0.400±0.003 | 119 | 3.2 | 7.5 |
