# Supplementary material for: YOLO-MDEW:Improved YOLOv8 for application of wood board edge banding defect detection
Source: PLoS One. 2026 May 8;21(5):e0348758. doi: 10.1371/journal.pone.0348758 (PMC13155551; doi:10.1371/journal.pone.0348758)
Supplement: S8 Table — (DOCX) [file pone.0348758.s018.docx]

S8 Table. Comparison between RT-DETR and YOLO-MDEW.

|  | **Model** | **P** | **R** | **mAP50** | **mAP50:95** | **FPS** | **Params (M)** | **GFLOPs** |
| --- | --- | --- | --- | --- | --- | --- | --- | --- |
|  | RT-DETR | 0.721 | 0.678 | 0.723 | 0.395 | 28 | 42 | 95 |
|  | YOLO-MDEW | 0.756 | 0.668 | 0.740 | 0.400 | 119 | 3.2 | 7.5 |
