# Supplementary material for: YOLO-MDEW:Improved YOLOv8 for application of wood board edge banding defect detection
Source: PLoS One. 2026 May 8;21(5):e0348758. doi: 10.1371/journal.pone.0348758 (PMC13155551; doi:10.1371/journal.pone.0348758)
Supplement: S9 Table — (DOCX) [file pone.0348758.s019.docx]

S9 Table. Scale-wise Recall for YOLOv8 and YOLO-MDEW.

|  | **Model** | **Small** | **Medium** | **Large** |
| --- | --- | --- | --- | --- |
|  | YOLOv8 | 65.6% | 74.1% | 67.3% |
|  | YOLO-MDEW | 67.8% | 76.8% | 72.9% |
