# Supplementary material for: YOLO-MDEW:Improved YOLOv8 for application of wood board edge banding defect detection
Source: PLoS One. 2026 May 8;21(5):e0348758. doi: 10.1371/journal.pone.0348758 (PMC13155551; doi:10.1371/journal.pone.0348758)
Supplement: S10 Table — (DOCX) [file pone.0348758.s020.docx]

S10 Table. Per-class Precision and Recall for YOLOv8.

|  | **Defect Category** | **Precision** | **Recall** | **mAP50** |
| --- | --- | --- | --- | --- |
|  | Glue seam | 56.1% | 54.5% | 52.8% |
|  | Board gap | 77.7% | 78.9% | 81.8% |
|  | Tape residue | 72.7% | 68.9% | 77.4% |
|  | Edge banding longer | 79.5% | 79.9% | 86.5% |
|  | Glue residue | 53.8% | 58.4% | 52.8% |
|  | Edge banding tackless | 81.8% | 75.0% | 84.4% |
|  | Short edge banding | 94.1% | 56.6% | 76.8% |
|  | Edge banding dirty | 63.2% | 67.5% | 66.4% |
