# Supplementary material for: YOLO-MDEW:Improved YOLOv8 for application of wood board edge banding defect detection
Source: PLoS One. 2026 May 8;21(5):e0348758. doi: 10.1371/journal.pone.0348758 (PMC13155551; doi:10.1371/journal.pone.0348758)
Supplement: S11 Table — (DOCX) [file pone.0348758.s021.docx]

S11 Table. Per-class Precision and Recall for YOLO-MDEW.

|  | **Defect Category** | **Precision** | **Recall** | **mAP50** |
| --- | --- | --- | --- | --- |
|  | Glue seam | 57.8% | 55.6% | 57.3% |
|  | Board gap | 80.7% | 77.6% | 79.9% |
|  | Tape residue | 70.9% | 71.7% | 75.8% |
|  | Edge banding longer | 81.4% | 83.8% | 87.4% |
|  | Glue residue | 55.5% | 56.4% | 57.6% |
|  | Edge banding tackless | 80.4% | 75.0% | 88.5% |
|  | Short edge banding | 92.9% | 58.3% | 76.5% |
|  | Edge banding dirty | 63.0% | 70.0% | 69.6% |
